# Emissions and exposures of graphene nanomaterials, titanium dioxide nanofibers, and nanoparticles during down-stream industrial handling

**DOI:** 10.1038/s41370-020-0241-3

**Published:** 2020-06-16

**Authors:** Karin Lovén, Sara M. Franzén, Christina Isaxon, Maria E. Messing, Johan Martinsson, Anders Gudmundsson, Joakim Pagels, Maria Hedmer, NanoLund, Karin Lovén, Sara M. Franzén, Christina Isaxon, Maria E. Messing, Anders Gudmundsson, Joakim Pagels, Maria Hedmer

**Affiliations:** 1 Ergonomics and Aerosol Technology, Lund University, SE-22100 Lund, Sweden; 2 Solid State Physics, Lund University, SE-22100 Lund, Sweden; 3 Medical Radiation Physics, Department of Translational Medicine, Lund University, SE-22100 Malmö, Sweden; 4 Occupational and Environmental Medicine, Lund University, SE-22100 Lund, Sweden

**Keywords:** Occupational exposure, Electron microscopy, Thermal-optical carbon analysis, Direct-reading instruments, PIXE, Aerosol

## Abstract

Today, engineered nanomaterials are frequently used. Nanosized titanium dioxide (TiO_2_) has been extensively used for many years and graphene is one type of emerging nanomaterial. Occupational airborne exposures to engineered nanomaterials are important to ensure safe workplaces and to extend the information needed for complete risk assessments. The main aim of this study was to characterize workplace emissions and exposure of graphene nanoplatelets, graphene oxide, TiO_2_ nanofibers (NFs) and nanoparticles (NPs) during down-stream industrial handling. Surface contaminations were also investigated to assess the potential for secondary inhalation exposures. In addition, a range of different sampling and aerosol monitoring methods were used and evaluated. The results showed that powder handling, regardless of handling graphene nanoplatelets, graphene oxide, TiO_2_ NFs, or NPs, contributes to the highest particle emissions and exposures. However, the exposure levels were below suggested occupational exposure limits. It was also shown that a range of different methods can be used to selectively detect and quantify nanomaterials both in the air and as surface contaminations. However, to be able to make an accurate determination of which nanomaterial that has been emitted a combination of different methods, both offline and online, must be used.

## Introduction

Due to their novel and valuable properties compared with bulk materials, the use of engineered nanomaterials is increasing. Graphene, a 2D carbon nanomaterial, has a robust, but also flexible, structure rendering it useful in a variety of applications [1]. It can exist in different structures such as graphene, graphene oxide, and graphene nanoplatelets [2, 3]. Its electrical and thermal properties makes graphene useful in for example transistors [2, 4] and chemical sensors [5], and the optical properties can be used in biological sensors [6]. Another application of graphene is as metal surface coatings to inhibit corrosion [7, 8] and to reduce wear and friction on sliding metal surfaces [9, 10].

Multiple reviews [11–15] have generally concluded that graphene toxicity depends on the physiochemical properties of the nanomaterial. However, one of the most widely recognized mechanism for graphene-nanomaterial-induced toxicity in living systems is the induction of oxidative stress and production of reactive oxygen species (ROS) [15]. In a more recent review by Fadeel et al. [16], the authors highlighted the need for standardized graphene characterizations, and of robust and validated toxicological assays in order to advance the field of graphene toxicity.

Studies of occupational exposure to nanomaterials are needed to make complete risk assessments. Basinas et al. [17] showed that many exposure assessments have been done for carbon nanotubes (CNTs), carbon nanofibers (CNFs), and titanium dioxide nanoparticles (TiO_2_ NPs), but far less for other engineered nanomaterials, such as graphene. Sanchez et al. [12] stated that there is a need for measurements of airborne graphene exposure levels at both research laboratories and full-scale manufacturing facilities. This was further pointed out by Arvidsson et al. [13], who requested that workplace emissions and exposures of graphene should be investigated. Only a few emission and exposure measurements during production [18–21] and handling [18] of graphene nanomaterials have since then been conducted. These studies showed low levels of exposures to graphene. However, according to Lee et al. [18], monitoring of other work tasks including down-stream graphene handling processes is needed for a full understanding of the exposure situation. To our knowledge, no studies of emission and exposure measurements during down-stream handling processes of graphene nanomaterials, such as manufacturing of ink and surface coatings containing graphene, have previously been conducted.

Another common nanomaterial is TiO_2_. It is used in paints and sunscreens [22, 23] as well as in transistors [24], biosensors [25], cancer treatment [26], and different surface coatings [27, 28]. Nanosized TiO_2_ is commonly found as spherical NPs, but can also be produced in other shapes including NFs [29] and nanowires [30]. The toxicity of nanosized TiO_2_, especially spherical ones, has been studied to a greater extent than graphene. As with graphene, the different physiochemical properties of TiO_2_ NPs have a strong influence on the toxicity [31]. Generally though, only moderate effects have been observed, including pulmonary inflammation [32–34] and pathological neural changes [35] after inhalation/instillation in rodents. Induction of DNA damage has also been observed in lung cell studies [36, 37]. A few studies have been carried out on exposed workers. Pelclova et al. [38] showed for example that the leukotriene levels in exhaled breath condensate were elevated in workers exposed to TiO_2_ NPs. The toxicity of TiO_2_ NFs has not been as thoroughly investigated. Hurbánková et al. [39] showed the development of serious lung inflammatory and cytotoxic processes after intratracheal instillation of TiO_2_ NFs in rats and Medina-Reyes et al. [40] observed cell cytotoxicity and genomic instability of TiO_2_ NF exposure on alveolar epithelial cells. In addition, Allegri et al. [41] performed a comparative exposure study on alveolar epithelial cells showing that TiO_2_ NFs were more toxic than TiO_2_ NPs.

Occupational airborne exposure to TiO_2_ NPs has been extensively studied. Production of TiO_2_ NPs, bagging and handling of the NP dry powder, as well as incorporation of the NPs into other products has been investigated. Different handling tasks, both with NP dry powder and NP containing liquid, have been shown to constitute an occupational exposure risk [42–45]. The review by Debia et al. [46] further strengthen this conclusion. However, studies of TiO_2_ NF exposure in occupational settings have, to our knowledge, not yet been conducted.

The Organisation for Economic Cooperation and Development (OECD) have suggested a harmonized three-tiered approach for nanomaterial emission and exposure assessments [47] and recommendations for measurement strategies and instrument use [48].

This study aims to generate new knowledge about emissions and exposures of nanomaterials not extensively studied previously. Emissions and exposures, with a focus on different graphene nanomaterials (both nanoplatelets and oxide) and different TiO_2_ nanomaterials (both NFs and NPs), were characterized with a multi-metric approach during down-stream industrial handling. Emission and exposures of carbon black (CB) and copper (Cu) were also measured in a few cases. Different sampling and aerosol monitoring methods were evaluated to be able to recommend methods to be used specifically for these nanomaterials to complement the different tiers and measurement strategies described by OECD. An additional aim was to assess the potential of secondary inhalation exposure, caused by resuspension of particles deposited on surfaces.

## Methods

### Facilities

Measurements were performed 2016 and 2017 at two different workplaces, with 20 and 15 employees respectively, hereafter “Study A” and “Study B”. As recommended by the OECD [47], initial contextual information was gathered (tier 1) and basic exposure assessments (tier 2) were performed at both workplaces prior to conducting the expert exposure assessment (tier 3) described herein.

During Study A, different nanomaterials including graphene nanoplatelets, spherical TiO_2_ NPs, CB, and Cu were handled. The nanomaterials were used in ink formulations for printing electronics, sensors, and labels. Measurements were conducted in a chemistry laboratory and a printing laboratory. In Study B, the nanomaterials handled included graphene oxide and TiO_2_ NFs for use in friction and wear reducing surface coatings. Measurements were conducted in a chemistry laboratory and a test laboratory. Both workplaces were equipped with general ventilation and process ventilation systems such as fume hoods. The amount of TiO_2_ handled during the two studies differed by three orders of magnitude (from about 5 kg per day during Study A to about 5 g per day during Study B). The amount of graphene nanomaterial handled was a few grams per day during both Study A and B. Similar processes were investigated during both studies.

### Work tasks

Different work tasks were performed during the two studies and a thoroughly written logbook documented the specific activities carried out. Table 1 shows these work tasks with detailed descriptions. In Study A, three workers performed the work tasks and in Study B only one.

**Table 1:** Work tasks performed at the two companies, types of engineering controls and types of personal protective equipment (PPE).

Work task number	Work task	Work task description	Location/ Engineering controls	PPE
Study A				
A1	Preparation of graphene nanoplatelets ink (weighing, mixing). Note that this is not the same ink printed with in A3!	Weighing of graphene nanoplatelet powder, addition of liquid and mixing.	Chemistry laboratory/Fume hood	Half-face respirator (A + P3), lab coat, protective gloves of nitrile, goggles
A2—day 1	Preparation of titanium dioxide nanoparticle ink (weighing, mixing)	Weighing of titanium dioxide nanoparticle powder, weighting of liquid. Addition of powder by sieving into the liquid and mixing.		
A2—day 2	Preparation of titanium dioxide nanoparticle ink (weighing, mixing)			
A3	Screen printing with graphene ink (HDPlas Graphene Ink), 10 sheets	Transfer of ink to the screen printer, start of printing process and a number of sheets was printed. The printed sheets were placed in the drying oven. After the printing process the ink was removed and the screen printer and its equipment was cleaned.	Printing laboratory/process ventilation	Clean room lab coat, hair net, protective gloves of nitrile
A4	Screen printing with carbon black ink (C740), 10 sheets			
A5	Screen printing with carbon black ink (CXT0641), 10 sheets			
A6—day 1	Screen printing with carbon black ink (C7102), 20 sheets			
A6—day 2	Screen printing with carbon black ink (C7102), 22 sheets			
A7	Screen printing with copper ink (CP-PLS-010715-R1A), 10 sheets			
Study B				
B1	Preparation of graphene oxide coating (weighing, mixing)	Weighing of graphene powder, addition of liquid to the powder and mixing. Transferring of the liquid to another container followed by addition of glass beads and more mixing. Finally, 2 steps of filtration of the liquid to remove the glass beads.	Chemistry laboratory/Parts in open fume hood (weighing, mixing), parts on open bench (filtration steps)	Half-face respirator (A + P3), lab coat, protective gloves of nitrile, goggles
B2	Spraying and curing graphene oxide coating, and cleaning/washing up after	Transfer of the liquid coating to a paint sprayer, cleaning of metal plates, paint spraying of metal plates, placing the coated plates in a furnace. Cleaning of paint sprayer in the spray booth followed by washing up mixing equipment in the fume hood and at the sink.	Test laboratory/parts in spray booth, parts in open fume hood and sink (cleaning and washing up)	
B3	Abrasion test with a metal brush on graphene oxide coating	The coated plate was placed in the abrasion testing equipment. A brush, rotated with a certain speed, was placed to have contact with the plate. When the surface was abraded the test was ended.	Test laboratory/none	Laboratory coat, protective gloves of nitrile, goggles
B4	Abrasion test with a nylon brush on graphene oxide coating			
B5	Preparation of titanium dioxide nanofiber coating (weighing, mixing)	Weighing of titanium dioxide nanofiber powder, addition of liquid to the powder and mixing. Transferring of the liquid to another container followed by addition of glass beads and more mixing. Finally, 2 steps of filtration of the liquid to remove the glass beads.	Chemistry laboratory/parts in open fume hood (weighing, mixing), parts on open bench (filtration steps)	Half-face respirator (A + P3), lab coat, protective gloves of nitrile, goggles
B6	Spraying and curing titanium dioxide nanofiber coating, and cleaning/washing up after	Transfer of the liquid coating to a paint sprayer, cleaning of metal plates, paint spraying of metal plates, placing the coated plates in a furnace. Cleaning of paint sprayer in the spray booth followed by washing up mixing equipment in the fume hood and at the sink.	Test laboratory/parts in spray booth, parts in open fume hood and sink (cleaning and washing up)	
B7	Abrasion test with a metal brush on titanium dioxide nanofiber coating	The coated plate was placed in the abrasion testing equipment. A brush, rotated with a certain speed, was placed to have contact with the plate. When the surface was abraded the test was ended.	Test laboratory/None	Laboratory coat, protective gloves of nitrile, goggles
B8	Abrasion test with a nylon brush on titanium dioxide nanofiber coating			

### Engineering controls and personal protective equipment

Different types of exposure control techniques and enclosures, as well as different types of personal protection equipment (PPE), were used during the different work tasks (Table 1).

### Sampling strategy

**Fig. 1:**
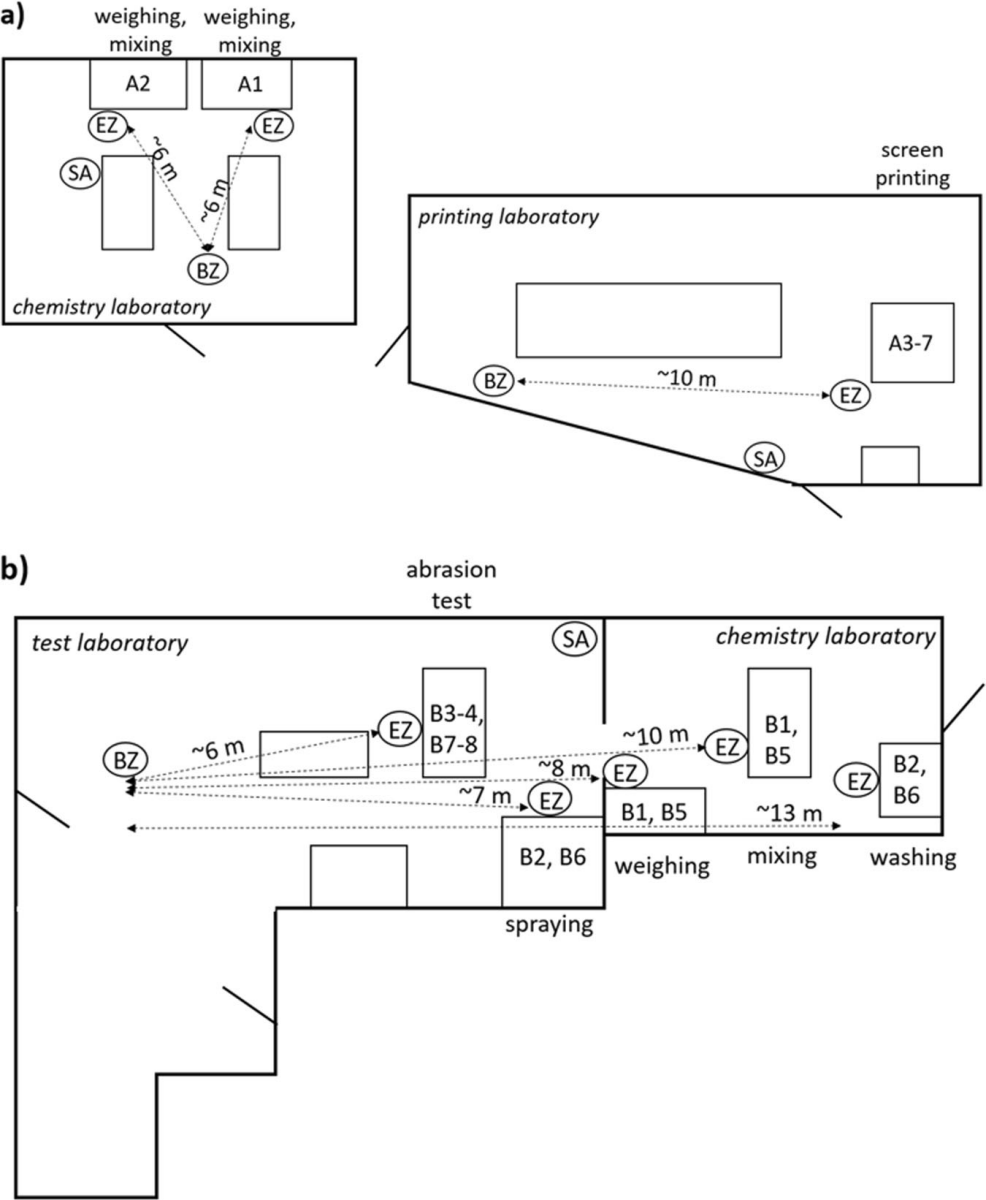
Schematics of the facilities. The location of where the different work tasks were performed, and the placement of the different measurement zones, emission zone (EZ), background zone (BZ), and supply air (SA), are shown during **a** Study A and **b** Study B.

Time resolved and filter based measurements of airborne particles were conducted in four different spatial zones, a methodology described in detail in Isaxon et al. [49]. The measurement zones included: (1) emission zone (EZ)—no more than a few centimeters from a potential source, (2) personal breathing zone (PBZ)—within a radius of 30 cm from a worker’s nose and mouth, (3) background zone (BZ)—at least 2–3 m away from any potential particle source, and (4) supply air (SA)—in the inflowing air from the general ventilation system. Figure 1 shows the schematics of the facilities in Study A and B including where the different work tasks were performed and where the different measurement zones were located.

### Air sampling methods and analyses

#### Filter sampling for elemental composition

Time-integrated emission zone, personal breathing zone, and background zone samples were collected by open-face sampling on 25-mm filters mounted in filter cassettes. Quartz filters (SKC Inc., USA) were used to collect samples for elemental carbon (EC) analysis. Filters made of polycarbonate (SKC Inc., USA) were used for analysis of titanium (Ti), and of cellulose (pore size 0.45 µm, SKC Inc., USA) for elemental analysis of Cu. Pumps (Escort ELF, MSA, USA) with a flow rate of 2.3 l/min for the polycarbonate filters and 3.0 l/min for the quartz and cellulose filters—checked before and after sampling—were used for all sample collection.

Quantification of EC of the graphene nanomaterials was conducted according to the NIOSH NMAM 5040 protocol for thermal-optical analysis (DRI Model 2001 OC/EC Carbon Analyzer, Atmoslytic Inc., USA) [50]. Temperature steps for the EC fraction were: 680 °C (EC1), 750 °C (EC2), and 900 °C (EC3). The method was modified with an extended oxidation time, 150 s instead of 30 s, at the highest temperature, 900 °C, in order to achieve complete oxidation of all carbonaceous nanomaterials [51]. The limit of detection (LOD) for EC was determined to be 0.06 µg C/cm^2^ (corresponding to 4-h sampling with a carbon airborne concentration of 0.5 µg/m^3^).

Quantification of Ti was conducted by Particle-Induced X-ray Emission (PIXE) analysis [52]. In PIXE, a 2.55 MeV proton beam is focused on the filter specimen. This renders the atoms in a state of high excitation, which causes inner shell vacancies. The characteristic X-ray emission lines are caused by the quickly occurring transition to a state of lower energy. When protons are used, the cross section for the creation of an inner shell vacancy is very high, thereby the sensitivity is very high; for instance, the LOD for Ti was <6 ng/cm^2^.

The elemental quantification of Cu was performed by digestion with 1 ml concentrated nitric acid (Nitric Acid, Trace metal grade, Fisher Chemicals) in an oven (60 °C) for 16 h, followed by dilution to 10 ml with Milli-Q water to a stock solution. Analysis was performed by inductively coupled plasma-mass spectrometry (ICP-MS, iCAP Q, Thermo Scientific, Germany). The LOD for Cu was three times the standard deviation of blank filters i.e. <0.01 µg/sample. All results were blank filter corrected.

#### Filter sampling for SEM analysis

To be able to morphologically characterize the workplace air, regarding engineered NPs and NFs and their aggregates and agglomerates, during down-stream handling processes, total dust fraction on filters according to Nilsson et al. [53] and Vaquero et al. [21] were collected. Time-integrated samples were collected at 2.3 l/min (sampling pump Escort ELF, MSA, USA) by open-face sampling on 25-mm polycarbonate filters (pore size 0.4 µm, SKC Inc., USA). The filters were analyzed by Scanning Electron Microscopy (SEM) using a Hitachi SU8010 Cold Field Emission SEM (Hitachi, Japan) with an acceleration voltage of 10 kV. A sputtering tool (Q150T ES, Quorum, UK) was used to coat the sample with 10 nm of platinum:palladium (Pt:Pd, 80:20). A minimum of 10 random 1.25 × 10^−5^ cm^2^ areas of the filter were imaged and used for quantifying the number of particles. During Study A, 17 filter samples were collected and the highest LOD was determined to be 0.47 cm^−3^. During Study B, 13 filter samples were collected and the highest LOD was determined to be 1.91 cm^−3^. In addition to the areas imaged for particle quantification, a larger part of the surface was investigated with lower resolution in order to identify any engineered nanomaterial.

#### Direct reading instruments

Several different direct-reading time resolved instruments were used in the four different measurement zones (Table 2). The aerodynamic particle diameter size distribution in the range 0.5–20 µm was obtained by two Aerodynamic Particle Sizers (APS, model 3321, TSI Inc., USA) with a time resolution of 5 s. Two condensation particle counters (CPC, model 3775 and 3010, respectively, TSI Inc., USA) were used to measure the total number concentration of particles > 0.007 µm, with a time resolution of 1 s. One APS and one CPC measured in the emission zone, while the second APS and CPC were measuring in the background zone. An aethalometer (model AE33, Magee Scientific, USA) was used to measure the black carbon (BC) mass concentration (as a proxy for EC) in the background zone, with a time resolution of 1 min. During Study A, the measurements in the emission zone were supplemented with a fast aerosol mobility size spectrometer (DMS Model 500 MkII, Cambustion, UK) and a DustTrak (model DRX 8533, TSI Inc., USA). The DMS measured the particle number size distribution in the size range 0.005–1 µm, with a time resolution of 1 s. The DustTrak measured the particle mass concentration in four size fractions: PM_1_, PM_2.5_, respirable and PM_10_, with a time resolution of 1 s. During Study B, a portable aethalometer (model AE51, AethLabs, USA) was used to measure the BC mass concentration in the emission zone, with a time resolution of 10 s.

**Table 2:** Direct reading instruments used for time resolved studies of particle emissions.

	Personal breathing zone	Emission zone	Background zone	Supply air
Study A	Partector, portable aethalometer	APS, CPC, DMS, DustTrak	APS, CPC, Aethalometer	P-Trak, DustTrak
Study B	Partector, portable aethalometer	APS, CPC, portable aethalometer	APS, CPC, Aethalometer	P-Trak, DustTrak

During both studies, two instruments were carried by the workers to measure in the personal breathing zone. A similar second portable aethalometer to measure the BC mass concentration, with a time resolution of 10 s, and an aerosol dosimeter (Partector, Naneos, Switzerland) to measure the lung deposited surface area (LDSA) concentration, with a time resolution of 1 s.

For particle measurements in the supply air, a P-Trak (model 8525, TSI Inc., USA) and a DustTrak (model DRX 8534, TSI Inc., USA) were used to assess the particle number concentration (0.02–1 µm) and the particle mass concentration (0.1–15 µm), respectively, with a time resolution of 1 s.

### Surface sampling method and analysis

During Study B, tape samples from different surfaces were collected according to a tape stripping method described by Hedmer et al. [54]. Tape samples were collected at the end of the workdays from surfaces in the near-field zone of the exposure source (<1 m). Surface contaminations in the far-field zone (>1 m), including two offices and a conference room, were also studied (Table 4). Two field blank tape samples were also obtained. The tape samples were prepared and analyzed with the same SEM method as the air filter samples.

## Results

After the completion of tier 1 (contextual information gathering) and tier 2 (basic exposure assessment), we concluded that tier 3 expert exposure assessments were needed at both companies. The tier 3 measurements are the ones described herein.

### Filter sampling

Table 3 shows the results from the filter samples collected during work tasks using CB, graphene nanomaterials and TiO_2_ NPs and NFs. Note that some of the filters have been sampled for more than one work task (according to Table 1). During the one work task where Cu was used (A7), no concentration of Cu was detected in either the personal breathing zone or the emission zone.

**Table 3:** Results from the filter based measurements during the work tasks using graphene nanoplatelets, graphene oxide, titanium dioxide (TiO_2_) nanofibers (NFs) and nanoparticles (NPs) and carbon black (CB).

Work task number	Nanomaterials handled during the work task	Sampling time (min)	SEM analysis		Elemental carbon (µg/m^3^)	Metal conc. Ti (µg/m^3^)
			Detection of nanomaterial (Yes/No)/Type	Number conc. (cm^−3^)		
Study A (2016)						
Personal breathing zone						
A1	Graphene nanoplatelets	42	^a^	^a^	<LOD	–
A2—day 1	TiO_2_ NPs	83	Yes/TiO_2_ NPs	25	–	7.5
A2—day 2	TiO_2_ NPs	45	Yes/TiO_2_ NPs	1.8	–	2.1
A3-6—day 1	CB, graphene	106	Yes/CB	^a^	5.6	–
A6—day 2	CB	39	–	–	<LOD	–
Emission zone						
A1	Graphene nanoplatelets	43	Yes/graphene nanoplatelets	^a^	26	–
A2—day 1	TiO_2_ NPs	83	Yes/TiO_2_ NPs	25	–	70
A2—day 2	TiO_2_ NPs	57	Yes/TiO_2_ NPs	7.0	–	28
A3	Graphene	13	^a^	^a^	<LOD	–
A4	CB	15	Yes/CB	^a^	<LOD	–
A5	CB	15	Yes/CB	^a^	98	–
A6—day 1	CB	31	Yes/CB	^a^	8.2	–
A6—day 2	CB	37	–	–	<LOD	–
Study B (2017)						
Personal breathing zone						
B1–4	Graphene oxide	172	No	<LOD	1.3	–
B5–6	TiO_2_ NFs	100	Yes/TiO_2_ NFs	^a^	–	<LOD
B7-8	TiO_2_ NFs	60	No	<LOD	–	<LOD
Emission zone						
B1–2	Graphene oxide	207	Yes/graphene oxide	^a^	1.9	–
B3	Graphene oxide	32	No	<LOD	<LOD	–
B4	Graphene oxide	111	^a^	^a^	<LOD	–
B5	TiO_2_ NFs	54	Yes/TiO_2_ NFs	^a^	–	2.2
B6	TiO_2_ NFs	129	No	<LOD	–	<LOD
B7	TiO_2_ NFs	23	No	<LOD	–	<LOD
B8	TiO_2_ NFs	123	No	<LOD	–	0.2
Background zone						
B1–4	Graphene oxide	480	No	<LOD	0.2	
B5–6	TiO_2_ NFs	202	No	<LOD	–	<LOD
B7–8	TiO_2_ NFs	173	No	<LOD	–	<LOD

–Not sampled.

^a^Not possible to determine.

### Graphene detection and quantification

**Fig. 2:**
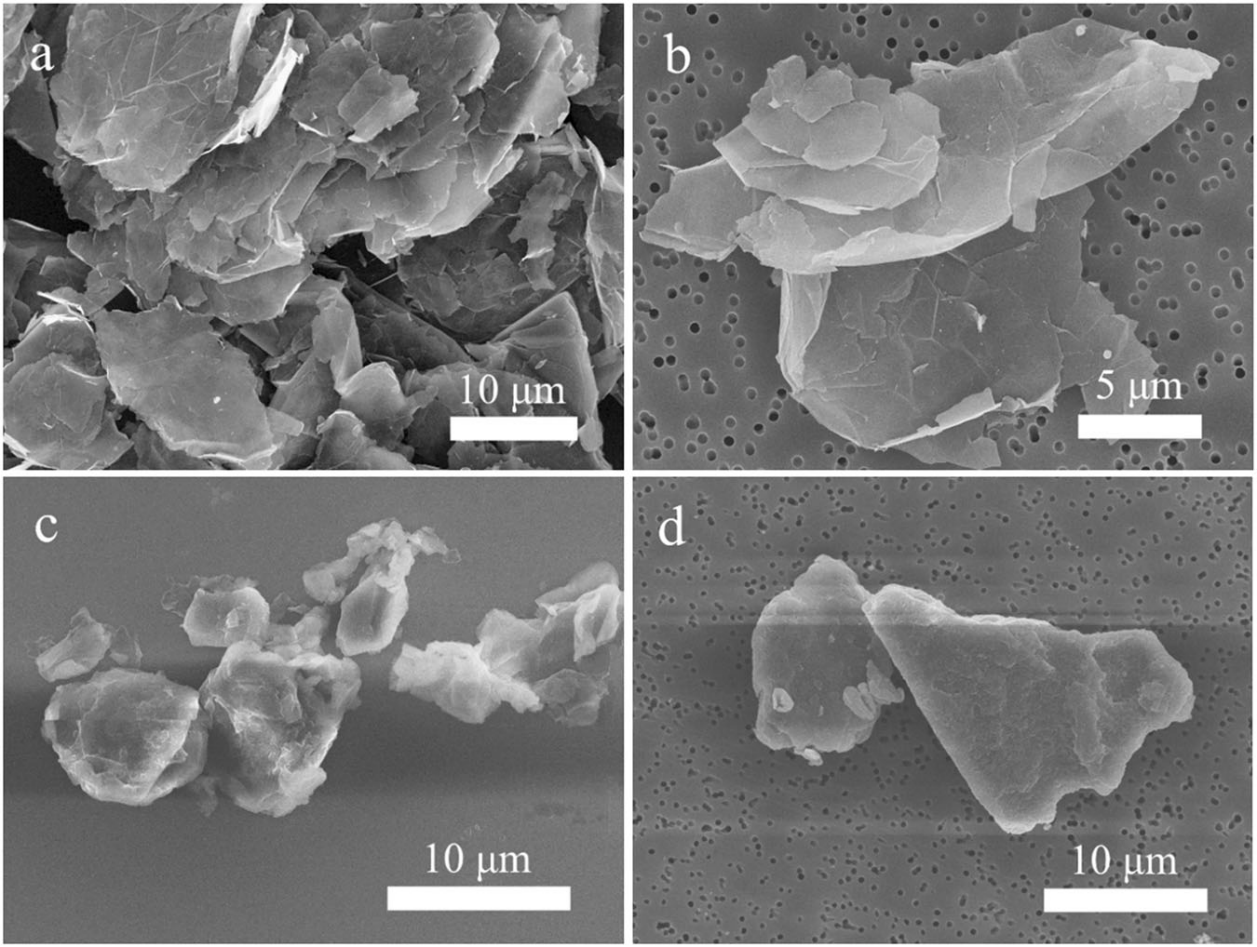
SEM images of the different graphene raw materials and the materials measured in the emission zones. **a** The raw material of graphene nanoplatelets used during Study A, **b** graphene nanoplatelets found on the emission zone filter sampled during work task A1 during Study A, **c** the raw material of graphene oxide used during Study B, and **d** graphene oxide found on the emission zone filter sampled during work task B1–2 during Study B.

**Fig. 3:**
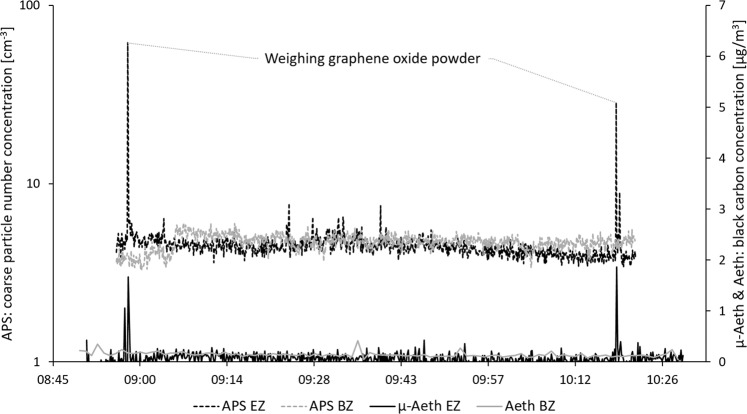
Coarse particle number concentration (APS, left *y*-axis) and black carbon concentration (portable aethalometer, µ-Aeth & aethalometer, Aeth, right *y*-axis) measured in the emission zone (EZ) and the background zone (BZ). The figure shows the measurements during weighing of the graphene oxide powder during Study B (work task B1). Note the different scales on the *y*-axes.

**Fig. 4:**
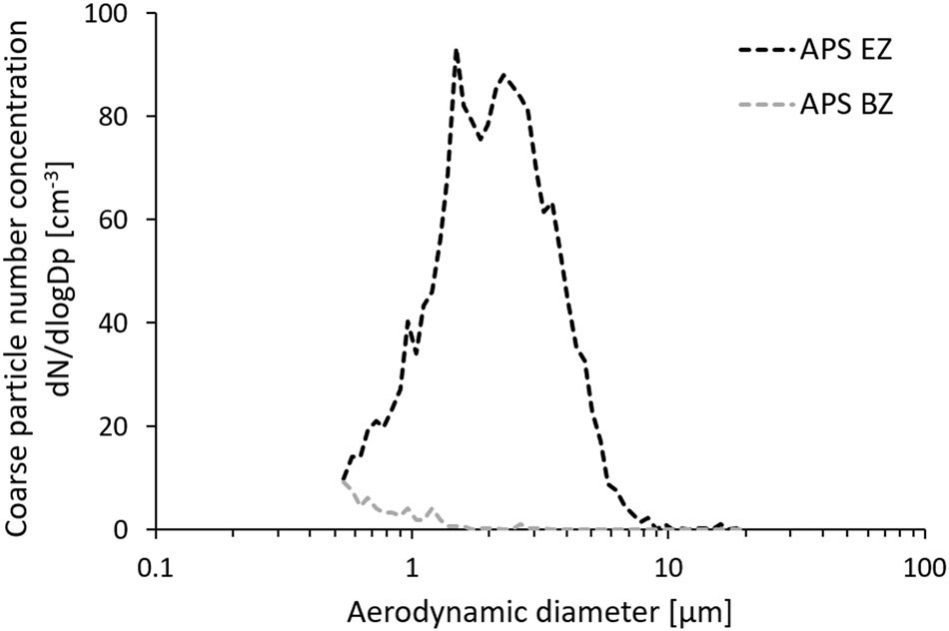
Aerodynamic particle size distribution measured in the emission zone (APS EZ) and the background zone (APS BZ) in Study B. The figure shows the size distribution of the initial peak in Fig. 3.

Table 3 shows that graphene nanomaterials were detected (Fig. 2) on the emission zone filters at both workplaces. Figure 2a shows the raw graphene nanoplatelet material used during Study A, and Fig. 2b shows the sampled material found in the emission zone during handling of graphene nanoplatelet powder (work task A1). Figure 2c shows the raw graphene oxide material used during Study B, and Fig. 2d shows the sampled material found in the emission zone during handling of graphene oxide powder (work task B1). The amounts of graphene nanomaterial in the emission zones (26 and 1.9 µg/m^3^ for Study A and B, respectively) were quantified as EC with thermal-optical analysis. During Study B, EC (most likely from graphene oxide) was also quantified in the personal breathing zone (1.3 µg/m^3^). Two of the direct reading instruments (APS and portable aethalometer) showed clear particle concentration peaks in the emission zone during the 1-min weighing event of graphene oxide powder (performed twice) during Study B (work task B1), see Fig. 3. The coarse particle number size distribution (count median aerodynamic diameter (CMD) of ~2 µm) of the initial graphene oxide particle concentration peak (seen in Fig. 3) is shown in Fig. 4. From the SEM images (Fig. 2c, d), the size of the graphene oxide particles is estimated to be about 10–20 µm.

### Titanium dioxide nanofiber detection and quantification

**Fig. 5:**
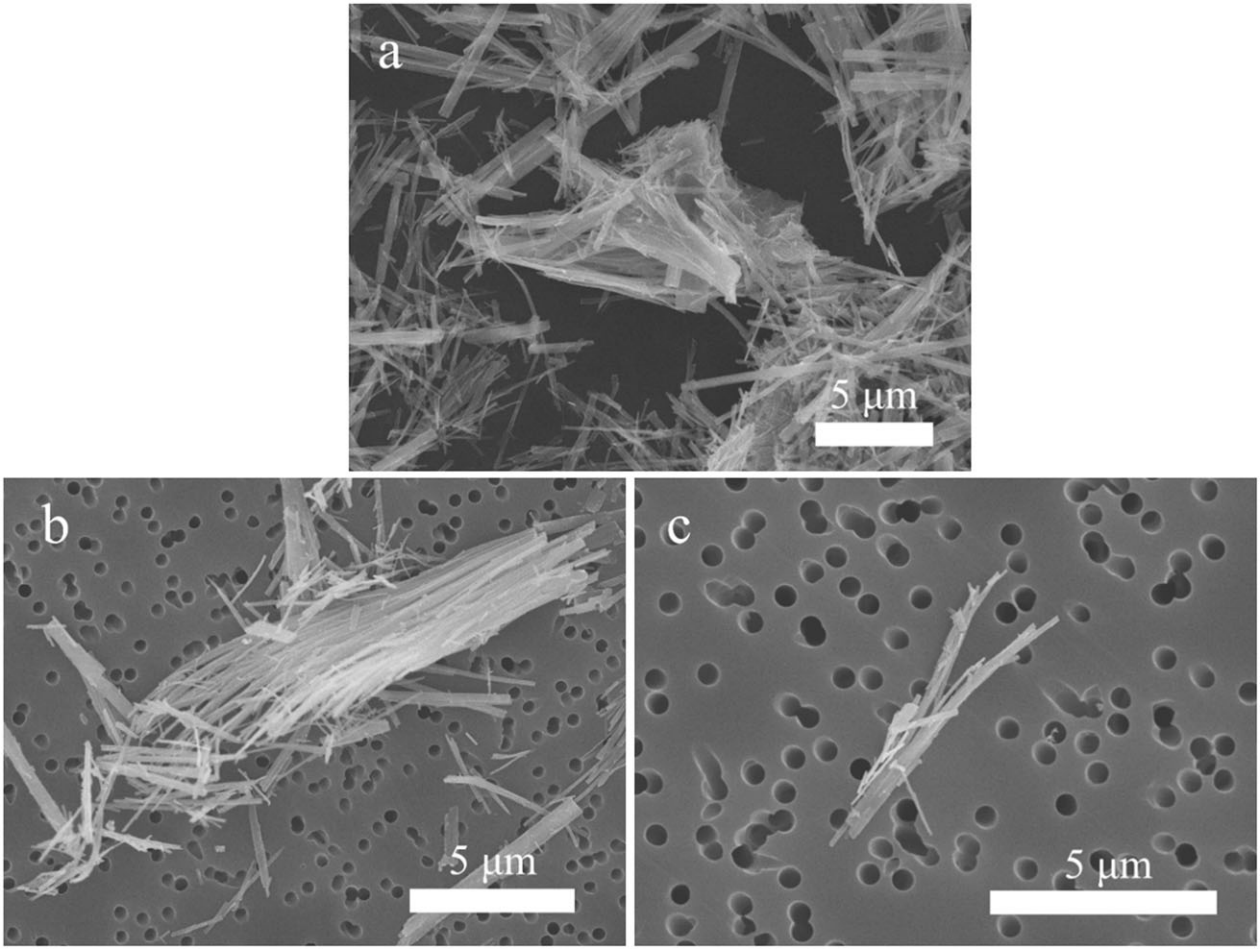
SEM images of the TiO_2_ NF raw material and the material measured in the emission zone and the personal breathing zone in Study B. **a** The raw material of TiO_2_ NFs used, **b** TiO_2_ NFs found on the emission zone filter sampled during work task B5, and **c** TiO_2_ NFs found on the personal breathing zone filter sampled during work task B5–6.

**Fig. 6:**
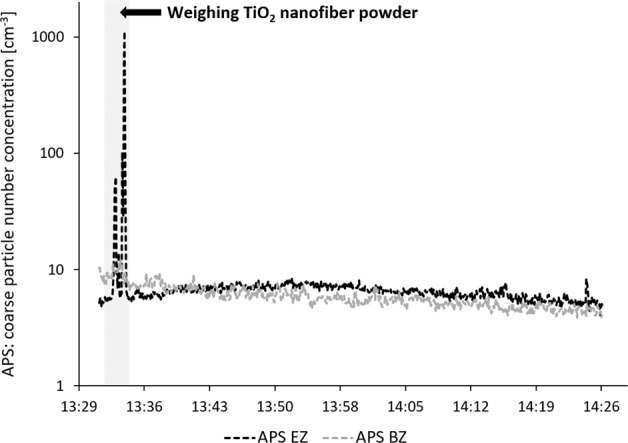
Coarse particle number concentration measured in the emission zone (APS EZ) and the background zone (APS BZ). The figure shows the measurements during the 2-min weighing of the TiO_2_ NF powder during Study B (work task B5).

As shown in Table 3, TiO_2_ NFs, handled at the workplace during Study B, could be detected (Fig. 5) both in the emission zone and in the personal breathing zone during powder handling (work task B5). Figure 5a shows the raw TiO_2_ NF material used during Study B, and Fig. 5b, c shows examples of sampled material found in the emission zone and personal breathing zone, respectively. The sampled fiber length was assessed to be 1–15 µm. TiO_2_ NF concentration in the emission zone was 2.2 µg/m^3^, quantified as Ti with PIXE. The APS in the emission zone showed particle concentration peaks during the two-minute weighing of the TiO_2_ NF powder, see Fig. 6. The coarse particle number size distribution of the concentration peaks seen in Fig. 6 revealed a CMD of ~0.7 µm (not shown).

### Titanium dioxide nanoparticle detection and quantification

**Fig. 7:**
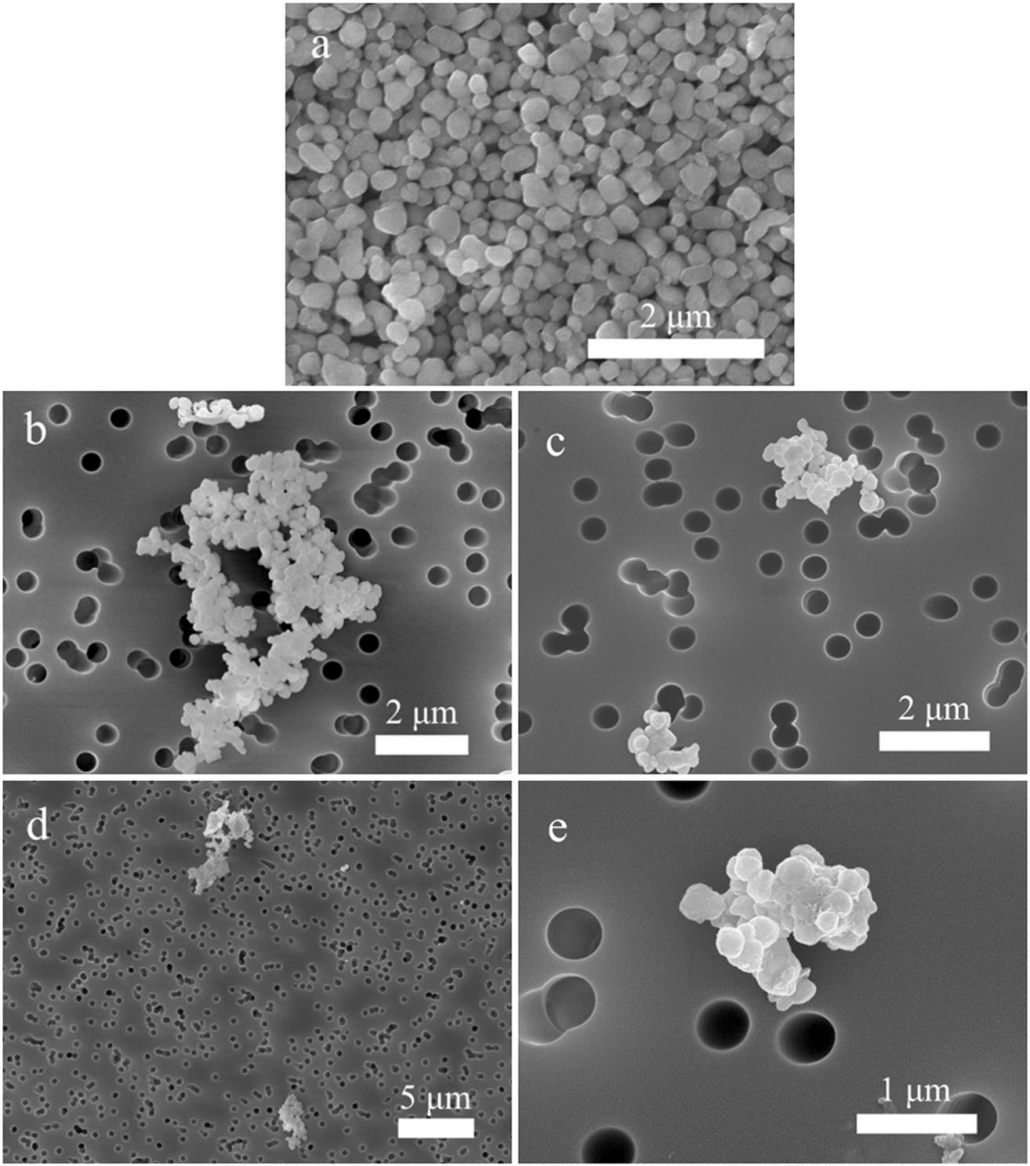
SEM images of the TiO_2_ NP raw material and the material measured in the emission zone and the personal breathing zone  in Study A. **a** The used raw material of titanium dioxide NPs, **b** TiO_2_ NPs found on the emission zone filter sampled during work task A2—day 1, **c** TiO_2_ NPs found on the emission zone filter sampled during work task A2—day 2, **d** TiO_2_ NPs found on the personal breathing zone filter sampled during work task A2—day 1, and **e** TiO_2_ NPs found on the personal breathing zone filter sampled during work task A2—day 2.

**Fig. 8:**
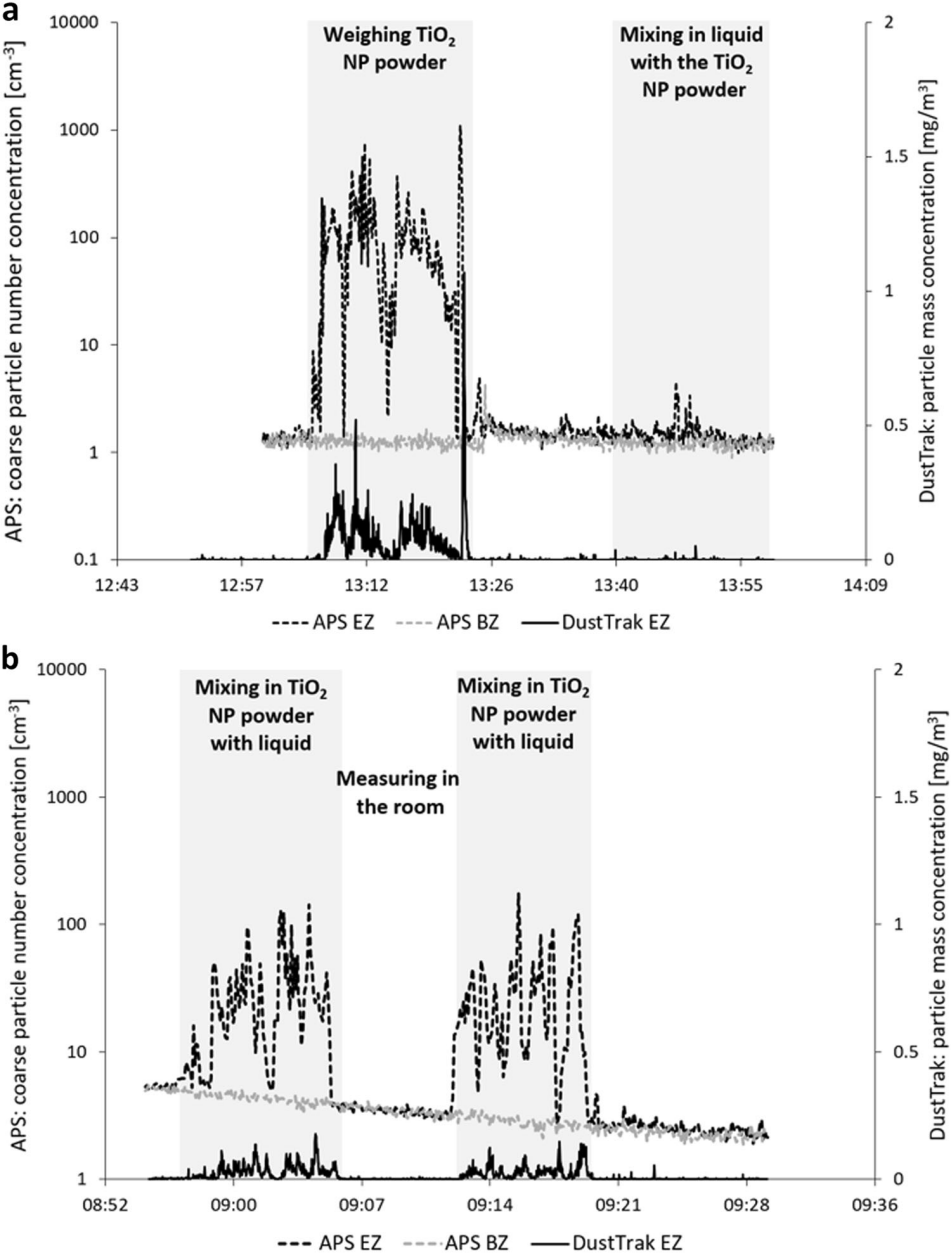
Coarse particle number concentration (APS, left *y*-axis) and particle mass concentration (DustTrak, right *y*-axis) measured in the emission zone (EZ) and the background zone (BZ). The figure shows the measurements during weighing and mixing of the TiO2 NP powder during Study A. **a** Shows work task A2—day 1 and **b** shows work task A2—day 2.

Spherical TiO_2_ NP agglomerates, handled at the workplace during Study A, were detected (Fig. 7) on filters from both the emission zone and the personal breathing zone during handling of the powder on two separate days (work task A2—day 1 and A2—day 2). The concentration reached 70 and 28 µg/m^3^ in the emission zone for the first and second day, respectively (quantified with the PIXE), and 7.5 and 2.1 µg/m^3^ in the personal breathing zone the first and second day, respectively. Particle concentration peaks during weighing and mixing of the TiO_2_ NP powder were detected by APS and DustTrak in the emission zone during both the first and second day, see Fig. 8. Similar to the PIXE results, the particle concentrations measured with the APS and DustTrak were lower during the second day. The Partector in the personal breathing zone showed a particle exposure reaching a peak lung deposited surface area concentration of 92 µm^2^/cm^3^ during the first day and of only 9 µm^2^/cm^3^ the second day (not shown).

### Surface contamination

**Fig. 9:**
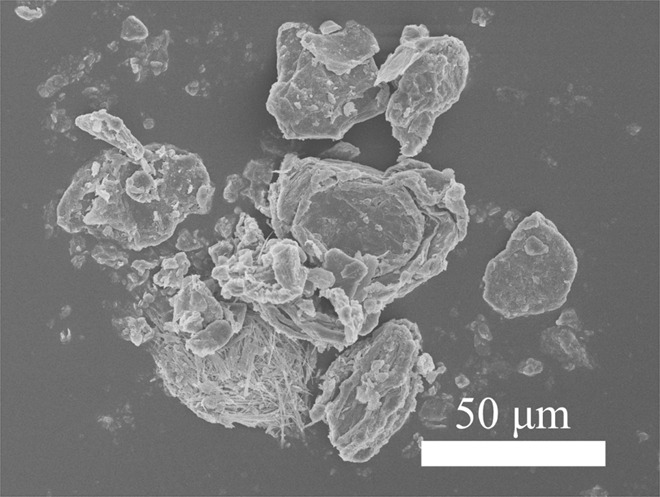
SEM image of TiO_2_ NFs and graphene oxide. The image shows materials found as surface contamination at the sink in the chemical laboratory in Study B.

Workplace surfaces in the chemical laboratory, test laboratory, offices, and conference room were tape sampled in Study B. In total, 15 tape samples were collected, and TiO_2_ NFs were detected in 20% (*n* = 3) and graphene oxide in 20% (*n* = 3) (Table 4). Surface contamination of both TiO_2_ NFs and graphene oxide was found on only one surface, at the sink in the chemical laboratory which was related to work task B1–2 and B5–6. A SEM image of the nanomaterial-related surface contamination can be seen in Fig. 9. The length of the TiO_2_ NFs was assessed to be 10–50 µm. TiO_2_ NFs were also detected on the plate with TiO_2_ NF based coating after the abrasion test with the nylon brush. No surface contamination of nanomaterial was found outside the chemical and test laboratories.

**Table 4:** An overview of the 15 tape-sampled surface locations and the presence of titanium dioxide nanofibers and graphene oxide particles on different surfaces found during Study B.

Sampling location	Room	Related to work task number	Surface characteristics		SEM analysis	
			Material	Assessed indication of roughness	Detection of TiO_2_ nanofiber (Yes/No)	Detection of graphene oxide particles (Yes/No)
Work areas						
Work areas in fume hood	Chemistry laboratory	B1, B5	Stainless steel	Smooth	No	No
Cover on work area		B1, B5	Cardboard	Smooth	No	No
Sink		B1–2, B5–6	Stainless steel	Smooth	Yes	Yes
Desk next to abrasion testing machine	Test laboratory	B3–4, B7–8	Laminate	Smooth	No	Yes
Floors						
Floor in front of fume hood	Chemistry laboratory	B1, B5	Epoxy coated concrete	Smooth	No	No
Floor in front of spray booth	Test laboratory	B2, B6	Epoxy coated concrete	Smooth	No	No
Floor	Conference room	–	Parquet	Smooth	No	No
Floor	Office 1	–	Laminate	Smooth	No	No
Door sill	Office 2	–	Wood	Smooth	No	No
Other surfaces						
Balance in fume hood	Chemistry laboratory	B1, B5	Plastic	Smooth	No	Yes
Cover under the brush in the abrasion testing machine	Test laboratory	B3–4, B7–8	Metal	Smooth	Yes	No
Plate with graphene based coating, sampled after abrasion test with metal brush		B3	Metal with graphene based coating	Smooth	No	^a^
Plate with graphene based coating, sampled after abrasion test with nylon brush		B4	Metal with graphene based coating	Smooth	No	^a^
Plate with TiO_2_ nanofiber based coating, sampled after abrasion test with metal brush		B7	Metal with TiO_2_ nanofiber based coating	Smooth	No	No
Plate with TiO_2_ nanofiber based coating, sampled after abrasion test with nylon brush		B8	Metal with TiO_2_ nanofiber based coating	Smooth	**Yes**	No

–Far-field surfaces.

^a^Not possible to determine which type of particles that are detected .

## Discussion

This study aimed to characterize workplace emissions and exposure of graphene nanoplatelets, graphene oxide, TiO_2_ NFs, and NPs, as well as CB and Cu during industrial handling. The results showed that powder handling, regardless of nanomaterial, generates the highest (among the investigated processes) particle emissions and exposures. It was shown that a range of different methods successfully can be used to selectively detect and quantify nanomaterials both in the air and on surfaces, and that, to be able to make an accurate determination of which nanomaterial that has been emitted when, a combination of different methods, both offline and online, must be used.

### Measurements of airborne graphene nanomaterials

The results show that graphene can be detected and quantified with several different methods. With the offline method consisting of filter sampling and SEM analysis we were, by comparing with the original nanomaterial, able to identify both graphene nanoplatelets used during Study A, and graphene oxide used during Study B (Fig. 2). Lee et al. [18] and Vaquero et al. [21] have also reported similar graphene-like structures in workplace air samples. According to OECD [48], both SEM and TEM are frequently used for determination of nanomaterial present in the workplace air.

In Fig. 4, the peak particle number size distribution during handling of graphene oxide powder (seen in Fig. 3) showed a CMD of ~2 µm. In the SEM images of both collected graphene nanoplatelets and graphene oxide, larger particles were found (10–20 µm). This is not contradictive since 2D materials such as graphene may have an aerodynamic diameter much smaller than its geometrical dimensions [55], a fact that indicates its ability to reach the alveolar region of the lung when inhaled.

The EC concentration (assumed to originate from graphene) was quantified with thermal-optical analysis (OC/EC) of filter samples. EC analysis is much less labor intensive than the SEM/TEM method. In previous workplace studies of graphene, Lee et al. [18] and Vaquero et al. [21] also used EC as an exposure metric for graphene, while exposure data of EC are missing in the studies by Spinazze et al. [19] and Boccuni et al. [20]. In the current study, EC concentrations collected as total dust fractions were detected both in emission zone and personal breathing zone filter samples, for example during weighing and mixing of graphene oxide. Lee et al. [18] collected respirable fractions of EC while Vaquero et al. [21] collected total dust fraction, but none of these studies detected any airborne EC concentrations during manufacturing of graphene nanomaterial.

We used on-line measurements of equivalent BC (eBC) as a proxy for EC by using a portable aethalometer. This allowed for highly time-resolved measurements and identification of exposure peaks for several work tasks. The combination of OC/EC analysis and portable aethalometer for graphene quantification has previously been used by Lee et al. [18] at a graphene nanoplatelets manufacturer. They found the clearest emission peaks in terms of eBC during graphene weighing. We have shown that these methods can be used also at down-stream handling facilities. The portable aethalometer and the APS (both time-resolved instruments) were used together with a thoroughly written logbook. By this approach we could determine that weighing and mixing the dry powder material generated the highest particle emission and exposure levels. The importance of documenting the activities to be able to match the measured concentration profiles have previously been demonstrated by e.g., Ham et al. [44], Hedmer et al. [51], and Isaxon et al. [49].

Both OC/EC analysis and eBC measurements of graphene nanomaterials is subject to cross sensitivity by other carbonaceous nanomaterials such as CB and CNTs, but also from soot particle sources, such as diesel exhaust, that may be present in workplace air. Both EC and eBC concentrations were substantially elevated in the emission zone compared with simultaneous measurements in the background zone during Study B, suggesting that cross sensitivity to background EC or eBC was low.

The eBC mass concentration was reported assuming the standard instrument settings. This may have affected the accuracy of the method as an offset of the results if the ENMs have an altered instrument response, for example by a different mass absorption cross section compared with the standard value that is based on atmospheric soot. This uncertainty can be tolerated when it comes to ENM exposures, where the aim often is to link exposure peaks with specific work tasks. In the literature, an eBC/EC ratio of 0.14 was found during simulated powder handling of CNTs [56].

Even though a combination of offline and online methods is recommended, different methods can be used individually for specific needs, depending on what type of information that is needed. For graphene, the following can be used: (1) SEM analysis for accurate identification of the nanomaterial, (2) EC analysis of filter samples for an accurate assessment of higher carbon concentrations (relatively high LOD), (3) direct reading portable aethalometer for highly time-resolved eBC assessment, and (4) direct reading APS for low-concentration (although less specific) detection of particle emissions (low LOD). This means that with access to one of these methods, a preliminary emission and exposure assessment should be possible to conduct.

### Measurements of airborne titanium dioxide nanofibers and nanoparticles

NFs are another type of nanomaterials that are of concern, especially if they are long (>5 µm) and insoluble, due to their potential to cause adverse health effects, including frustrated phagocytosis and accumulation over time finally causing lung disease [57]. During Study B, TiO_2_ NFs were detected both in the emission zone and the personal breathing zone with the offline filter sampling method followed by SEM analysis (Fig. 5). They appear to be present in the workplace air as bundles, and some of the fibers detected by SEM during Study B were at least 5 µm and could therefore constitute a risk if inhaled. Bianchi et al. [58] have recently shown that long (around 10 µm) TiO_2_ NFs induce cell cytotoxicity in vitro and inflammation in vivo, while shortened (around 2 µm) TiO_2_ NFs seem to mitigate the toxic effects, even without macrophages present in the in vitro cultures. SEM-analysis can also be used to quantify the particle concentration in the air. However, it is a time consuming process, and during Study B it was shown to be difficult to distinguish the different fibers due to agglomeration and therefore to count them. The PIXE analysis was a valid alternative, even though it gives the concentration as a different metric (mass concentration, µg/m^3^). Detection and quantification can also be conducted with direct reading measurements (APS could be used to determine what specific handling process generated the particles (Fig. 6); weighing the dry powder material), but to confirm fiber emissions, time-integrated filter sampling followed by SEM is necessary.

General NP dry powder handling has previously been shown to constitute a potential source for worker exposure by e.g., Huang et al. [42] and Curwin and Bertke [43]. Lee et al. [59] found substantial total mass concentrations during powder collection of TiO_2_ NPs in the pigment industry (500–5000 µg/m^3^), however, no elemental analysis of Ti was carried out. In the current study, this was confirmed, were elevated concentrations of TiO_2_ NPs were found in both the emission zone and the personal breathing zone (with SEM, PIXE, APS, DustTrak, and Partector) during weighing and mixing of dry TiO_2_ NP powder. Interestingly, the mass concentrations of Ti (measured with PIXE) were about a factor 10 lower in the personal breathing zone than in the emission zone. This reduction factor was most likely achieved by the use of engineering controls in the emission zone (fume hood) and such a factor could be used for other assessments of personal exposure where only measurements in the emission zone can be conducted or vice versa.

Another observation was that the particle concentrations measured with PIXE, APS, DustTrak, and Partector were all lower during the second day compared with the first day of weighing and mixing of the TiO_2_ NP powder. As shown in Fig. 8, the procedure of mixing the powder with the liquid had been adjusted from the first to the second day. During the first day, the TiO_2_ NP powder were weighed first and then the liquid was added to form the printing ink. The second day, this procedure had been adjusted so that the TiO_2_ NP powder were directly mixed in with the liquid. This clearly shows that with an easy change in the handling procedure, the particle emissions and exposures can be lowered by as much as a factor 10 (for example seen with the Partector where the lung deposited surface area went from 92 µm^2^/cm^3^ during the first day to only 9 µm^2^/cm^3^ during the second day). The Partector has also previously been shown to be an important tool for personal exposure assessments to improve workplace monitoring [60–62].

Quantification of Ti from TiO_2_ nanomaterials by ICP-MS is not possible due to that TiO_2_ is a poorly soluble oxide. Laser-ICP-MS could be one alternative [45] and ICP-OES (optical emission spectrometer) [42] another. The current study has highlighted PIXE as a valid and reliable method for titanium quantification, which has also been shown by Relier et al. [63].

### Surface contamination

Surface contamination of TiO_2_ NFs and graphene oxide was for the first time studied at down-stream handling processes. Surface contaminations of both TiO_2_ NFs and graphene oxide were found on one surface (sink) in the near-field zone in the chemical laboratory in Study B. The detected surface contamination can probably be related to washing of equipment after preparations of both types of coatings. The surface contamination of TiO_2_ NFs and graphene oxide could probably be resuspended into the workplace air, which if so would cause a risk of secondary inhalation exposure. The percentage of TiO_2_ NF surface contamination in the collected tape samples were lower compared with what was previously found in a similar study [54]. This indicates that there was no widespread nanomaterial contamination at workplace B. An interesting finding was that TiO_2_ NFs could be detected on a tape sample from one of the abrasion-tested metal plates with TiO_2_ NF based coating. During the abrasion tests, dust containing manufactured nanomaterials is generated and could be emitted to the workplace air, especially since this process was openly performed at the workplace without any engineering controls.

### Proposed occupational exposure limits

Mihalache et al. [64] have reviewed the proposed occupational exposure limits (OELs) for a number of different nanomaterials. For TiO_2_ NPs, a range between 17 and 2000 µg/m^3^ have been proposed, with probably the most established one by NIOSH of 300 µg/m^3^ as an 8 h average airborne exposure [65]. The highest TiO_2_ concentration in the personal breathing zone during the current study was 7.5 µg/m^3^, corresponding to an 8 h average of 1.3 µg/m^3^, well below the suggested OEL. For TiO_2_ NFs, there is no specific OEL, but the BSI [66] suggested OEL for fibrous nanomaterials of 0.01 fibers/cm^3^ could be a suitable OEL to consult. However, in the current study it was found to be difficult to distinguish the different fibers by SEM due to agglomeration and therefore challenging to count them. The detected fibers were, furthermore, only found during the low resolution scanning-to-identify investigation and none were found during the quantification imaging, rendering the fiber number concentration below the LOD. Tsang et al. [67] demonstrated that a probabilistic approach can be used in the risk assessment of exposure scenarios involving production of TiO_2_ NPs. Thus, they could identify one out of seven exposure scenarios with statistically significant level of risk.

Neither for graphene nanomaterials are there any legal binding OELs yet, but e.g., Lee et al. [68] have based on data from a subchronical inhalation toxicity study calculated a recommended OEL for graphene nanomaterial to be 18 µg/m^3^. Also, health based guidance value for occupational inhalation exposure to graphene nanoplatelets was estimated to 212 µg/m^3^ by Spinazze et al. [69]. However, so far there are only few in vivo inhalation exposure studies reported in the literature. Thus, according to Pelin et al. [3] OEL for graphene cannot be determined based on the available data. The highest EC concentration in the personal breathing zone, in the current study, was 5.6 µg/m^3^, corresponding to an 8 h average of 1.2 µg/m^3^, well below the suggested OELs.

### Recommendations for occupational hygienists

As a general recommendation for occupational hygienists, the portable aethalometer could be one option for a small and easy-to-use instrument for personal exposure monitoring of carbon-based nanomaterials. However, from the current study, data from the personal sampling portable aethalometer could not be used due to too much noise, most likely arising from the short sampling time. This could possibly be addressed by longer averaging times [70] as well as noise reduction treatment during the data post-processing [71]. As a stationary instrument in the emission zone, the portable aethalometer worked well and can be an alternative to bulkier, more expensive and advanced instruments. For non-carbon-based nanomaterials, a suitable personal exposure instrument option would instead be a lung deposited surface area instrument. However, when it is critical with an accurate determination of which nanomaterial that is emitted, SEM analysis must be performed as a complement to the direct reading measurements. Open-face filter sampling was used in the current study to morphologically characterize the real emission and exposure situations of all released NPs and NFs and their aggregates and agglomerates. Chemical analysis is also a good option, especially when aiming to compare the result with existing OEL, if one can ensure that no other sources of that chemical compound are present in the workplace. For carbon-based materials (such as graphene) filter sampling with following EC analysis can be used. In the current study, open-face filter sampling was used, but it could have been possible to instead use a respirable cyclone to get the respirable EC concentrations. Then, it would be possible to compare measured EC concentrations with the recommended exposure limit of respirable EC set to 1 µg/m^3^ as an 8 h time weighted average concentration for CNTs and CNFs [72]. For TiO_2_ nanomaterials, filter sampling with following PIXE analysis would be the recommended chemical analysis to perform.

## Conclusions

Down-stream industrial handling of graphene nanomaterials and TiO_2_ NFs were, for the first time, investigated for particle emissions and exposures into the workplace air. We showed that weighing the dry powder material generated particle emissions, even though the exposure levels were low compared with proposed OELs. Surface contaminations of both TiO_2_ NFs and graphene were found on the sink in the chemical laboratory at workplace B. A range of different sampling and aerosol monitoring methods was used and evaluated. For a fast and reliable workplace emission and exposure assessment of graphene and TiO_2_ nanomaterials, a combination of different methods, both offline and online, must be used to ensure the detected emissions contain the specific nanomaterials. If a first exposure assessment should be performed (according to tier 2 of the OECD guidelines), access to only one or a few of these methods is enough. When a tier 3 exposure assessment is needed, a combination of multiple of the mentioned methods should be used together with additional direct reading instruments such as CPC and APS.
